
JOURNAL INFORMATION
==============================
NLM Title Abbreviation: Gastroenterol Rep (Oxf)
Journal ID: Gastroenterol Rep (Oxf)
NLM Journal ID: 101620508
Journal ID: gastro

Gastroenterology Report

EISSN: 2052-0034
Publisher: Oxford University Press

ARTICLE INFORMATION
==============================
PMCID: PMC4863195
DOI: 10.1093/gastro/gow014
Article ID: gow014
Article version: 1
Subjects: Chinese Abstracts

Chinese Abstracts – Volume 4 Issue 2*

*This article abstract was originally published in English. This translation has not been verified and should not be relied upon—it is provided for reference purposes only. The Publishers have not checked this translation and accept no liability for completeness or accuracy of this translation or the use of this translation for whatever purpose. This translation may be incomplete and inaccurate in whole or in part. If you need to rely upon a translation of this abstract, a professional human translator should be engaged to supply an accurate translation of the original English. When referencing articles from this journal, please always refer to the original English version, rather than a translated equivalent.

Print publication date: 2016 May
Electronic publication date: 2016 May 10
Volume: 4
Issue: 2
First page: C1
Last page: C3
Copyright: © The Author(s) 2016. Published by Oxford University Press and the Digestive Science Publishing Co. Limited.
Copyright year: 2016
License: This is an Open Access article distributed under the terms of the Creative Commons Attribution License (http://creativecommons.org/licenses/by/4.0/), which permits unrestricted reuse, distribution, and reproduction in any medium, provided the original work is properly cited.
License URL: https://creativecommons.org/licenses/by/4.0/

Page count: 3

==============================
OXFORD

*Gastroenterology Report*, 2016, 1–3

doi: 10.1093/gastro/gow014

### Chinese Abstracts – Volume 4 Issue 2*

### Review （综述）

### Should perioperative immunonutrition for elective surgery be the current standard of care?
### 围手术期免疫营养应该成为择期手术的标准治疗方案吗？

[摘要] 术后感染性并发症的出现将延长住院时间, 增加治疗费用, 同时推高了围手期并发症发生率。目前临床上采取了许多措施来预防或减少术后感染的发生， 如避免术前长期禁食、术后早期经口进食、代谢控制及术后早期活动等。尽管如此， 对于当前这个以降低术后并发症治疗费用为目标的医疗体系来说， 术后感染仍然是一个重大挑战。术前营养状况被认为是术后并发症的一项独立影响因素。应用肠内或肠外营养进行围手术期营养支持已被证实可以减少术后并发症， 尤其是对于具有高营养不良风险的患者。最近， 免疫营养对预防术后感染性并发症的作用得到了广泛研究。免疫营养制剂被发现可以调控术后免疫抑制及炎性反应, 在降低术后感染性并发症、缩短住院时间方面具有较高的性价比。在这篇综述中， 我们评价了围手术期免疫营养对于临床结局的积极作用及其成本效用, 并探讨了目前的临床应用指南及未来的临床应用价值。

[关键词] 免疫营养；感染；术后并发症；成本效用；指南

### Imaging panorama in postoperative complications after liver transplantation

### 肝移植术后并发症的影像学诊断全景

[摘要] 肝脏是第二常见的移植器官, 仅次于肾脏, 显然, 肝脏疾病是全球范围内一种常见且严重的疾病。随着外科学、肿瘤学及影像学技术的发展， 原位肝移植已经成为许多终末期肝病患者的一线治疗方案。超声和多普勒彩超是评估肝移植术后液体积聚和血管并发症最经济、性价比最高的影像学方法。 CT 可用以进一步证实超声检查结果, 并可用于肺部并发症的诊断。磁共振成像可用于发现肝管并发症、胆漏及神经系统并发症。本文论述了对于肝移植术后各种可能的并发症， 如何进行影像学技术的选择。

[关键词] 肝移植；术后并发症；影像学

### Mycetismus: a review　蕈中毒：综述

[摘要] 尽管很罕见, 但暴露于鹅膏蕈碱对人体健康有很大的影响, 其罕见性也给临床医生的诊断也带来了挑战， 医疗工作者很少对这一疾病进行报道。蕈中毒目前并无特定的解药。或许是因为罕见， 又或者是缺少关注，美国在蕈中毒的诊断、治疗及研究方面落后于欧洲近 30 年。因此, 美国有必要成立一个中心培训机构, 并加强相关研究和数据收集工作, 从而为蕈中毒患者提供更好的治疗。

[关键词] 蕈中毒；鹅膏蕈碱；蘑菇中毒；鹅膏毒素；维生素 C；西咪替丁；水飞蓟宾；N-乙酰半胱氨酸；肝移植；Ganzert 标准；King’s College 标准；Clichy 标准

### Original article（论著）

### Trends in malignant intraductal papillary mucinous neoplasm in US adults from 1990 to 2010: a SEER database analysis
### 美国 1990—2010 年间胰腺恶性导管内乳头状黏液腺瘤的趋势：SEER 数据分析

[摘要] 背景：导管内乳头状黏液腺瘤（IPMN）是一种癌前病变, 可很好地反映腺瘤演进。尽管 IPMN 恶性转化的风险已有很多研究, 但对于恶性 IPMN 患者的长期生存和相关预后因素等数据仍然十分有限。

方法：检索 SEER 数据库, 基于病理诊断或影像学证据来筛选出恶性 IPMN 病例。计算中位生存期和年龄调整的发病率。采用 Cox 比例风险模型来明确独立预后因素。

结果：基于 SEER 数据库, 1990—2010 年间有 2651 例患者被诊断为恶性 IPMN 。年龄调整的恶性 IPMN 发病率由 1990 年的 0.361/10 万（95% CI：0.285　0.451）逐步降至　2010　年的　0.135/10　万（95%　CI：0.098　0.186）。共计 564 例（21.3%）患者接受了外科手术治疗, 但手术例数亦呈下降趋势（从 1990—1995 年间的 132 例降至 2006—2010 年间的 96 例）。中位总生存期为 4 月, 且在 1990—2000 年间保持相对稳定。手术治疗可以明显降低死亡风险（HR＝0.45, 95% CI：0.40 ～0.53, P<0.001）。

结论： 1990—2010 年间, 恶性 IPMN 发病率明显下降, 但长期生存方面并无明显改善。其间仅有少部分病例接受了外科治疗, 提示恶性 IPNM 的长期生存是有进一步改善空间的, 而提高手术比例或是关键。

[关键词] 导管内乳头状黏液腺瘤；恶性转化；发病率；长期生存

*This article abstract was originally published in English. This translation has not been verified and should not be relied upon—it is provided for reference purposes only. The Publishers have not checked this translation and accept no liability for completeness or accuracy of this translation or the use of this translation for whatever purpose. This translation may be incomplete and inaccurate in whole or in part. If you need to rely upon a translation of this abstract, a professional human translator should be engaged to supply an accurate translation of the original English. When referencing articles from this journal, please always refer to the original English version, rather than a translated equivalent.
本文摘要最初是以英文发表。中文摘译未获认证，谨供参考。出版社没有对中文摘译进行核验，不负责译文的完整性和准确性，也不会将其用以其他目的。中文摘译可能会出现部分不完整或不准确的情况。如果你需要准确获悉摘要，最好能请一位专业译者针对英文原文进行翻译。阅读杂志文章，请参考英文原文，不推荐中文翻译。

### Practice pattern of ileal pouch surveillance in academic medical centers in the United States
### 美国学术型医疗中心回肠储袋的随访模式

[摘要] 目的：对于回肠储袋是否要进行监测、何时监测、如果监测,目前并未达呈共识。本研究旨在了解专家对储袋监测的看法以及储袋监测的实践模式, 并评估储袋监测能否提高瘤变的发现率。

方法：通过检索 MEDLINE 数据库的文献和美国克罗恩病/溃疡性结肠炎协会的名单, 找出可能关注过储袋患者的医生, 对其进行问卷调查。

结果：来自 32 家三级医疗机构的 52 名医生按要求完成问卷调查。41 名（79%）医生认为储袋镜监测是必要的, 36 名（69%）医生相信储袋镜+活检有利于瘤变的发现。但这些医生对于储袋镜的监测频率存在较大差异。 18 名（35%）医报告了在过去 5 年所发现的 4 例储袋腺瘤和 15 例储袋癌。这 18 名发现储袋瘤的医生在储袋镜检查时活检取材数反而更少（每个部位活检取材>3 者：44% *vs* 82%，$P$=0.005）。多因素分析显示, 每年随访的患者数量是影响储袋瘤检出率的唯一独立因素（OR＝1.5, 95% CI：1.1 ~2.1, $P$= 0.005）。

结论：大多数医生都认为应对储袋患者进行储袋镜检查加活检, 以监测储袋瘤变， 但对于最佳的监测频率仍有较大差异。储袋瘤的检出率似乎与医生手中的病例数及医生经验有关。

[关键词] 回肠储袋肛管吻合；储袋瘤变；结直肠切除术；溃疡性结肠炎

### Effective teaching of endoscopy: a qualitative study of the perceptions of gastroenterology fellows and attending gastroenterologists
### 有效的内镜教学：基于胃肠主治医生及专科培训医生观点的定性研究

[摘要] 背景：从胃肠专科培训医生（学习者）及主治医生（教学者）的角度来探讨何为有效的内镜教学， 目前尚鲜见报道。我们力求找出一些内镜教学的通用方案, 并了解其对培训医生及专科医生的影响。

方法：针对胃肠专科培训医生和主治医生, 就内镜教学资源、教学技术及改进方法进行小组讨论和调查。对由培训医生所选出的“最佳”内镜教师进行访问， 了解其内镜教学情况、教学方法、关键信息点及对内镜课程的看法。

结果： 19 名（68%）专科培训医生参加了美国胃肠内镜学会一年级专科培训医生的内镜课程, 他们认为该课程是有帮助的。 13 名（46%）培训医生进行了内镜模拟训练, 但其中位使用时间仅 1 小时。 5 个内镜培训项目仅 2 项采用了正式的内镜技能评估工具, 而且这 5 个项目都没有一个容易为培训医生所理解的内镜课程。培训医生认为他们最好的内镜学习方法就是实践操作， 他们同时也愿意主治医生采用不同的教学方法对其进行培训， 并认为对主治医生教学进行指导或有利于内镜教学。10 名（77%）主治医生接受了高级课程的培训, 但没有人接受如何教学的正规培训, 他们也都认为这样的培训会有帮助。

结论：标准化的内镜课程可能会有助于胃肠专科培训医生, 他们更倾向于通过操作来学习内镜， 但他们也想得到明确且有针对性的指导。无论是主治医生还是专科培训医生都认为， 对主治医生如何教学进行培训是有助于内镜教学的, 显示出“培训教师”课程的必要性。

[关键词] 内镜课程；内镜教学；胃肠专科医生培训

### Perceptions of non-alcoholic fatty liver disease – an Asian community-based study
### 对于非酒精性脂肪肝病的认识——基于亚洲人群的研究

[摘要] 背景和目的：非酒精性脂肪肝病（NAFLD）是最常见的慢性肝病, 同时也是代谢综合征的一项危险因素。 NAFLD 在全球范围内的患病率基于地域差异在 5% ~30% 不等。尽管流行病学资料显示代谢相关性疾病的患病率在新加坡呈快速增长, 但对于该地区 NAFLD 的数据却十分有限。本研究旨在了解新加坡 NAFLD 的患病率及公众对该病的认识。

方法：本研究是一项横断面观察研究, 旨在评估 NAFLD 患者的人口统计学、人体测量学及临床资料， 研究对象来自于一项胃肠公众论坛的参会者。 NAFLD 的诊断基于超声学标准；代谢综合征的诊断则基于国际糖尿病联盟的指南。通过自行设计的调查问卷来了解大家对 NAFLD 这种疾病的看法。

结果：共有 227 名研究对象纳入研究, 其中 40% 被诊断为 NAFLD 。相对于那些非 NAFLD 人群, NAFLD者男性居多, 年龄更大, 体质指数更高, 腰围更宽, 患代谢性综合征的比例更高（均 $P$<0.05）。尽管有 71.2% 的研究对象之前听说过 NAFLD, 但其中仅 25.4% 认为自己具有 NAFLD 的风险。有无代谢风险因素者对 NAFLD 的认识差不多（$P$>0.05）。值得注意的是，75.6% 的具有代谢危险因素者，并不认为自己存在 NAFLD 风险。

结论：我们的研究显示 NAFLD 在新加坡具有较高的患病率, 包括在那些非肥胖人群中。考虑到人们对 NAFLD 的发生风险认识不足, 有必要加强公众教育, 以提高人们对 NAFLD 的认识。

[关键词] 非酒精性脂肪肝病；流行病学；认识；新加坡

### Colorectal cancer screening in African Americans: practice patterns in the United States. Are we doing enough?
### 非洲裔美国人结直肠癌筛查的实践模式：我们做得够吗？

[摘要] 背景：结直肠癌是一种常见的恶性肿瘤，同时也是美国人主要致死原因。筛查可以降低结直肠癌发病率和死亡率。非洲裔美国人患结直肠癌的风险持续增高, 推荐在 45 岁时进行首次结直肠癌筛查。本研究旨在评估 45 ~49 岁非洲裔美国人的结直肠癌筛查率。

方法：从 Explorys 国家数据库中筛选出 45 ~49 岁非洲裔美国人, 分析这些患者结肠镜、乙状结肠镜或粪便隐血等筛查情况， 并按性别和医疗保险状态进行分层分析。$P$<0.05 表示差异有统计学意义。

结果：共有 181 200 名应行结直肠癌筛查的非洲裔美国人纳入分析, 其中仅31 480 例（17.4%）患者接受了至少一次的筛查。大多数（66.7%）患者是通过结肠镜进行筛查。女性的筛查率较男性更高（17.8% *vs* 16.7%，$P$<0.01）。大部分（66.0%）接受筛查的患者有私人医疗保险。

结论：种族、性别和医疗保险都会对结直肠癌筛查率造成影响。非洲裔美国人的结直肠癌筛查工作仍然不太理想。公共卫生倡议、医疗记录提醒和增强医患交流是提高非洲裔美国人结直肠癌筛查率的关键。

[关键词] 结直肠癌；筛查；种族

### Knowledge about non-invasive diagnostic tests for varices in liver cirrhosis: A questionnaire survey to the Gastroenterology Branch of the Liaoning Medical Association, China
### 对肝硬化静脉曲张非侵入性诊断试验的认识：对辽宁省医学会消化病分会的问卷调查

[摘要] 背景和目的：由于上消化道内镜具有侵入性，对于肝硬化静脉曲张的非侵入性诊断试验已经在许多研究中广泛应用。我们向辽宁省医学会消化病分会的委员发放调查问卷， 了解其对肝硬化静脉曲张非侵入性诊断试验的认识。

方法：向辽宁省医学会第 9 届消化病分会全体 42 名委员发放调查问卷, 了解其对肝硬化静脉曲张非侵入性诊断试验的认识。这 42 名委员来自 13 座城市的 33 家医院。

结果： 97.6% （41/42）的调查对象完成了问卷调查，其中大多为主任医生（85.4%），从事消化疾病医疗工作超过 20 年（80.5%），就职于三级医院（97.6%）。 46.3% 的调查对象所在科室, 每年接诊肝硬化胃食管静脉曲张病例超过 200 例。 90.2% 的调查对象所在科室, 常规使用上消化道内镜来诊断胃食管静脉曲张。只有 6 名（15%）调查对象会在临床中经常采用非侵入性诊断试验。 30 名（75%）调查对象知道至少一项静脉曲张的非侵入性诊断试验。对非侵入性诊断试验认识的多少与其所在医院肝硬化静脉曲张的病例数（$P$=0.038）和是否应用上消化道内镜来诊断静脉曲张（$P$=0.022）有关。

结论：本次问卷调查结果显示, 肝硬化患者静脉曲张的非侵入性诊断试验在中国辽宁省甚少应用于临床实践。有必要通过进一步的多中心观察研究来找到可靠的非侵入性诊断指标。

[关键词] 肝硬化；静脉曲张；门脉高压；非侵入性诊断试验；问卷调查

### Effect of quality of bowel preparation on quality indicators of adenoma detection rates and colonoscopy completion rates
### 肠道准备质量对腺瘤检出率和结肠镜完成率的影响

[摘要] 背景和目的：充分的肠道准备对于安全有效的结肠镜检查是非常重要的。结肠镜检查的质量指标应达到至少 95% 的肠镜完成率和至少 25% （50 岁以上男性）或 15% （50 岁以上女性）的腺瘤检出率。本研究旨在探讨肠道准备对腺瘤检出率和肠镜完成率的影响。

方法：回顾性纳入 2008 年 1 月至 2009 年 12 月间接受结肠镜检查的病例。主要结局指标是腺瘤检出率和肠镜完成率（抵达盲肠）。

结果：共 2519 例患者纳入研究, 其中 1030 例（41.0%）肠道准备评价为优, 1145例（45.5%）评价为良, 240例（9.5%）中等, 104例（4.1%）评价为差。肠道准备中、差者肠镜完成率分别为 75.4% 和 72.1% ，明显低于肠道准备优、良者（分别为 99.9% 和 99.7% ，P<0.001），且前者肠镜完成率质量达标（95%）的比例也明显低于后者（P<0.001）。全组患者中肠镜准备优、良、中、差者腺瘤检出率分别为 24.2% 、 26.8% 、 32.1% 和 22.1% , 差异无统计学意义（P=0.06）, 各组腺瘤检出率均质量达标（男性 25% , 女性 15%）。其中女性肠道准备优、良者腺瘤检出率相对更高, 男性肠道准备优、良、中者腺瘤检出率相对更高（P<0.001）。多因素分析显示, 男性患者腺瘤检出率明显增高, 但肠道准备质量对腺瘤检出率并无影响。

结论：肠道准备中、差者肠镜完成率明显低于肠道准备优、良者, 但肠道准备质量对腺瘤检出率并无影响。

[关键词] 肠道准备；腺瘤检出率；肠镜完成率；质量指标

### Case report（病例报告）

### A case of cerebral venous sinus thrombosis associated with Crohn's disease: dilemma in management
### 克罗恩病合并脑静脉窦血栓形成一例：治疗困境

### Gastric hyperplastic polyp with focal cancer
### 增生性胃息肉伴局灶癌

### A cause to consider for chronic unresolving diarrhea
### 慢性难治性腹泻的原因分析

### Radiation therapy-induced aortoesophageal fistula: a case report and review of literature
### 放疗引起的主动脉食管瘘：病例报告和文献复习

### Right paraduodenal hernia: report of two cases and review of literature
### 右侧十二指肠旁疝：两例报告和文献复习
